# Levodopa improved different motor symptoms in patients with Parkinson's disease by reducing the functional connectivity of specific thalamic subregions

**DOI:** 10.1111/cns.14354

**Published:** 2023-07-14

**Authors:** Wan Liu, Yang Shen, Yuan Zhong, Yu Sun, Jiaying Yang, Wenbin Zhang, Lei Yan, Weiguo Liu, Miao Yu

**Affiliations:** ^1^ Department of Rehabilitation The Affiliated Brain Hospital of Nanjing Medical University Nanjing China; ^2^ Department of Neurology The Affiliated Brain Hospital of Nanjing Medical University Nanjing China; ^3^ Department of Neurology Xiaogan Hospital Affiliated to Wuhan University of Science and Technology, The Central Hospital of Xiaogan Xiaogan China; ^4^ School of Psychology Nanjing Normal University Nanjing China; ^5^ Jiangsu Key Laboratory of Mental Health and Cognitive Science Nanjing Normal University Nanjing China; ^6^ International Laboratory for Children's Medical Imaging Research, School of Biological Sciences and Medical Engineering Southeast University Nanjing China; ^7^ Director of Joint Research Centre for University of Birmingham and Southeast University Southeast University Nanjing China; ^8^ Department of Public Health, School of Medicine and Holistic Integrative Medicine Nanjing University of Chinese Medicine Nanjing China; ^9^ Department of Functional Neurosurgery The Affiliated Brain Hospital of Nanjing Medical University Nanjing China

**Keywords:** functional connectivity, levodopa challenge test, Parkinson's disease, resting‐state functional magnetic resonance imaging, thalamus

## Abstract

**Background:**

The thalamus is an important relay station for the motor circuit of human. Levodopa can reverse the clinical manifestations by modulating the function of motor circuits, but its detailed mechanisms are still not fully understood. We aimed to explore (1) the mechanism by which levodopa modulates the functional connectivity (FC) in the subregions of the thalamus; (2) the relationship between the changed FC and the improvement of motor symptoms in Parkinson's disease (PD) patients.

**Methods:**

Resting‐state functional MRI was used to scan 36 PD patients and 37 healthy controls. The FC between the subregions in the thalamus and the whole brain was measured and compared under different medication states of PD patients. The correlation between the improvement of motor symptoms and changes in FC in the thalamus subregions was examined.

**Results:**

The PD on state exhibited decreased FC between the right pre‐motor thalamus and the right postcentral gyrus, as well as the right lateral pre‐frontal thalamus and the right postcentral gyrus. These decreases were positively correlated with the improvement of resting tremor. The PD on state also exhibited decreased FC between the left lateral pre‐frontal thalamus and right paracentral lobule, which was positively correlated with the improvement of bradykinesia.

**Conclusions:**

This study demonstrates that levodopa treats PD by decreasing the FC between the thalamus subregions and pre/post‐central cortex. Our results provide a basis for further exploration of the functional activity of thalamic subregions and offer new insights into the precision treatment in PD patients

## Graphical abstract

Parkinson's disease (PD) on state exhibited decreased functional connectivity (FC) between the right pre‐motor thalamus and the right postcentral gyrus, as well as the right lateral pre‐frontal thalamus and the right postcentral gyrus. These decreases were positively correlated with the improvement of resting tremor. The PD on state also exhibited decreased FC between the left lateral pre‐frontal thalamus and right paracentral loubule, which was positively correlated with the improvement of bradykinesia.

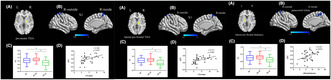

## 1 INTRODUCTION

Parkinson's disease (PD) is a progressive neurodegenerative disorder characterized by relative selective dopamine depletion in the midbrain substantia nigra and striatum. The main clinical motor symptoms of PD include bradykinesia, rigidity, and resting tremor. Currently, PD treatment is mainly symptomatic treatment, and levodopa is the most effective and widespread medication for managing PD.^1^ Levodopa treatment can improve the clinical manifestations with varying degrees of improvement by modulating the function of the basal ganglia‐thalamus‐motor cortex (BGMC) circuit. However, its detailed mechanisms are not fully understood.

As a relay station, the thalamus transmits information from peripheral sensory organs to the cortex and regulates sensory information processes, including somatosensory, visual, and auditory. Studies indicate that the thalamus plays an important and complex role in executive functions such as attention, behavioral flexibility, and goal‐directed behavior.^2^, ^3^, ^4^, ^5^ A previous study found that input from the thalamus triggers the activity of the primary somatosensory (S1) and visual (V1) cortex throughout the pre‐sensory period.^6^ Another fMRI study suggested that reduced thalamic‐PFC connectivity correlated with impaired working memory in schizophrenia patients.^5^ Moreover, the thalamus also plays an important role in the motor circuit in PD patients. Dirkx et al.^7^ proposed that the ventral intermediate nucleus of the thalamus (VIM) participated in the cerebellum‐thalamus circuit (tremor circuit) in PD. Helmich et al.^8^ illustrated that unlike VIM, the ventralis oralis posterior nucleus of the thalamus (VOP) is not directly involved in the tremor circuit. However, it receives a pathological signal from the basal ganglia and transmits it to the tremor circuit, leading to resting tremor.^7^ When dopamine is depleted in the striatum of BGMC circuit, the excitatory outflow of the ventral anterior nucleus (VA) and the ventrolateral nucleus (VL) in the thalamus decreases, leading to motor symptoms, including bradykinesia and rigidity in PD.^9^ Therefore, we can speculate that the dysfunction of different thalamus subregions may be involved in different motor circuits which generate the specific clinical symptoms in PD.

In addition, previous studies have reported that levodopa administration can act on the BGMC circuit and the tremor circuit to improve different clinical symptoms in PD.^10^, ^11^, ^12^ A neuroimaging study found that dopamine reduced the resting tremor symptom in PD patients by potentiating inhibitory mechanisms in a cerebellar nucleus of the thalamus.^7^ Another study found that levodopa can partially normalize the connectivity of the BGMC circuit, and the improvement of bradykinesia was associated with normalized connectivity of the striato‐thalamo‐cortical motor pathways.^12^ Most PD‐related motor circuit studies revealed the role of partial thalamus subregions in the pathogenesis of PD but have not systematically explored the function of each of the subregions. It is worth exploring whether the functional connectivity (FC) of other subregions in the thalamus changes under the acute levodopa challenge test and the association between these changes and improving clinical symptoms.

Currently, levodopa is still the most effective and basic drug in PD treatment. The application of levodopa can be translated into clinic effect immediately due to its short half‐life in PD patients and dopamine synthesis in the striatum, which depends on external levodopa.^13^ As a rapid and time‐saving test, the standardized acute levodopa challenge test can eliminate the interference of other confusing factors, such as the effects of other anti‐Parkinson's disease drugs and different medication times, and improve the accuracy of clinical diagnosis for Parkinson's disease. In addition, a good response to levodopa challenge test is an important predictor of good long‐term outcomes.^14^ Therefore, the acute levodopa challenge test is widely used in clinical diagnosis, treatment, and motor circuit study of PD.

In this study, we used the Brainnetome Atlas template to divide the whole thalamus into 16 subregions and applied resting‐state functional magnetic resonance imaging (rs‐fMRI) to investigate any significant changes in functional connectivity of each thalamus subregion under the effect of acute levodopa challenge test in PD patients. We hypothesized that (1) there are differential changes in functional connectivities in thalamus subregions after levodopa intervention and (2) the changed functional connectivities are correlated with improvements in specific clinical symptoms.

## 2 METHODS

### 2.1 Subjects

A total of 55 PD patients and 37 healthy controls (HCs) were recruited from the Affiliated Brain Hospital of Nanjing Medical University. An experienced neurologist diagnosed PD patients according to the United Kingdom Parkinson's Disease Society Brain Bank Clinical Diagnostic Criteria.^15^ To be included in the study, the PD patients had to have fulfilled the following: (1) the PD diagnosis fulfilled the UK Parkinson Disease Society Brain Bank Criteria for idiopathic PD, (2) the course of the disease was more than 1 year, (3) Anti‐parkinsonian medication was stable in the previous 3 months, (4) Mini‐Mental State Examination (MMSE) ≥ 24, (5) right‐handed, (6) aged between 50 and 75 years, and (7) vision or corrected vision and binaural hearing could meet the needs of evaluation and could be used to complete the examination. The requirements for HCs were as follows: (1) right‐handed, (2) aged between 50 and 75 years old, and (3) vision or corrected vision and binaural hearing could meet the needs of evaluation and could be used to complete the examination. The exclusion criteria of all subjects were as follows (1) history of disturbance of consciousness, (2) history of hereditary diseases, (3) history of schizophrenia, manic episode, and other mental diseases, (4) history of alcohol or drug dependence, (5) severe heart, liver, kidney, brain and hematopoietic system diseases, (6) contraindications of MRI scanning such as electronic and metal appliance implantation, and (7) T2‐weighted MRI showed cerebral infarction or vascular injury.

### 2.2 Study procedure

A standardized, acute levodopa challenge test^16^ was conducted in the fasting state, in both off and on levodopa conditions, and the Unified Parkinson's Disease Rating Scale (UPDRS)^17^ was assessed by an experienced neurologist. The UPDRS (motor section) was organized into separate tremor, rigidity, bradykinesia, and axial symptoms factors based on the findings of the most comprehensive factor analysis of the UPDRS (Table 1).

**TABLE 1:** Demographic and clinical characteristics of the sample. Values are mean (SD) unless stated otherwise.

	PD	HC	*t*/*z*/χ^2^	*p‐*value
Age (years)	61.0 (58.0, 65.0)^a^	59.0 (56.0, 63.0)^a^	−1.46	0.144
Sex (female/male)	17/19	18/19	0.02	0.903
Education	12.0 (9.0, 15.0)^a^	12.0 (9.0, 16.0)^a^	−0.33	0.739
MMSE	28.4 (1.6)	29.0 (1.5)	−1.61	0.112
Disease duration	6.3 (4.3)	NA	NA	NA
H and Y stage	2.2 (0.7)	NA	NA	NA

*Note*: Group differences in age and education using Mann–Whitney *U* test; Group differences in MMSE using independent sample *t* test; A chi‐square test was used to assess sex distribution.

Abbreviations: H and Y, Hoehn and Yahr staging; MMSE, Mini‐Mental State Exam; NA, not available.

^a^ The measurement data of skewed distribution are represented by median and quartile M (P25, P75).

PD patients were examined in the off state after withdrawing levodopa for at least 12 h and dopaminergic agonists for at least 24 h. They were assessed again after 1 h or when in a clinically on state after taking 1.5 times the usual morning levodopa equivalent of the daily dose (suprathreshold stimulation).^18^, ^19^ PD patients were scanned three times (“off” state, “on” state, and an intermediate time between “off” states and “on” states), whereas healthy control subjects were scanned only once. In this study, we included data for only two scanning times in the PD patients: on state and off state. The patients were also assessed on the H & Y staging scale^20^ and the MMSE (Mini‐Mental State Exam) scale while on their medication.

In this study, we selected 39 patients with a more than 30% (UPDRS III motor section) improvement rate on the levodopa challenge test. A total of 36 PD patients and 37 HCs were included in the final analyses after excluding 2 PD patients with poor magnetic resonance (MR) image quality and 1 PD patient with an unusually large head motion (see Preprocessing). The demographics and clinical details are shown in Tablea 1 and 2. The study was approved by the Medical Research Ethical Committee of the Affiliated Brain Hospital of Nanjing Medical University, and written informed consent was obtained from all participants.

**TABLE 2:** Clinical characteristics of the PD patients before and after levodopa intake. Values are mean (SD) unless stated otherwise.

Groups	PD off state	PD on state	*t/z*	*p*‐value
Total UPDRS‐III	32.5 (23.2, 40.8)^a^	17.0 (11.3, 20.8)^a^	−5.24	<0.001
Tremor	5.5 (3.0, 9.0)^a^	1.5 (1.0, 4.0)^a^	−4.81	<0.001
Rigidity	8.1 ± 4.1	3.7 ± 2.5	10.06	<0.001
Bradykinesia	12.8 ± 5.5	5.7 ± 3.3	11.36	<0.001
Axial symptoms	4.0 (3.0, 6.0)^a^	3.0 (2.0, 4.0)^a^	−3.94	<0.001

*Note*: UPDRS III total score, rigidity score, bradykinesia score between the PD off state and PD on state using paired *t*‐tests; Tremor score and axial symptoms score between the PD off state and PD on state using paired Wilcoxon signed rank test; UPDRS‐III was divided into subscores for tremor (UPDRS items 20 and 21), rigidity (UPDRS item 22), bradykinesia (UPDRS items 23–26 and 31) and axial symptoms (UPDRS items 27–30).

Abbreviations: UPDRS‐III, Unified Parkinson's Rating Scale motor section.

^a^ The measurement data of skewed distribution are represented by median and quartile M (P25, P75).

### 2.3 Data acquisition

MRI scanning was conducted using a 3 T MR scanner (Siemens, Verio, Germany). All subjects lay supine with their head fixed by foam pads with a standard birdcage head coil to minimize head movement.^21^ The participants were then instructed to remain as still as possible, close their eyes, remain awake, and not think of anything. Axial anatomical images were acquired using a T1 fluid‐attenuated inversion recovery sequence (repetition time [TR] = 2530 ms; echo time [TE] = 3.34 ms; flip angle [FA] = 7 degrees; matrix = 256 × 256; field of view [FOV] = 256 × 256 mm^2^; slice thickness/gap = 1.33/0.5 mm; 128 slices covered the whole brain) for image registration and functional localization. Functional images were subsequently collected in the same slice orientation with a gradient‐recalled echo‐planar imaging pulse sequence (TR = 2000 ms; TE = 30 ms; FA = 90 degrees; matrix = 64 × 64, FOV = 220 × 220 mm^2^; thickness/gap = 3.5/0.6 mm; in‐plane resolution = 3.4 × 3.4 mm; slice numbers = 31). A total of 140 volumes were obtained in this acquisition sequence, and each functional resting‐state session for each participant lasted 280 s.

### 2.4 Preprocessing

Data processing was performed using Data Processing & Analysis for (Resting‐State) Brain Imaging (DPABI 3.1, http://rfmri.org/dpabi) based on the Matlab2014b platform. The first 10 volumes of functional images for each subject were discarded. The remaining images were corrected by realignment to account for head motion, were normalized into the standard space using diffeomorphic anatomical registration through exponentiated Lie algebra (DARTEL), resampled to a 3 mm × 3 mm × 3 mm voxel size, had the nuisance variables regressed out, and spatially smoothed with a 6‐mm full width at half‐maximum (FWHM). The resulting fMRI data were band‐pass filtered (0.01 < *f* < 0.1 Hz) before proceeding to the next step. The nuisance variables included 24 motion parameters (six head motion parameters, six head motion parameters one time point before, and the 12 corresponding squared items), the signal‐averaged over the individual segmented cerebrospinal fluid (CSF) and white matter (WM) regions, and the linear and quadratic trends.^22^

Previous research found that the Friston‐24 covariates showed the greatest reductions in both positive and negative motion–blood‐oxygen‐level‐dependent (BOLD) relationships. In addition, the Friston‐24 approach produced the least motion‐related spikes when examining the BOLD signal after head motion correction.^23^ Subjects with a mean framewise displacement (FD) larger than three interquartile ranges from the sample median were defined as outliers and were excluded from further analysis to limit the impact of head motion.^24^

### 2.5 Functional connectivity

Sixteen subregions of the thalamus from the Human Brainnetome Atlas^25^ (http://atlas.brainnetome.org) were selected for this study, including the bilateral medial pre‐frontal thalamus, bilateral pre‐motor thalamus, bilateral sensory thalamus, bilateral rostral temporal thalamus, bilateral posterior parietal thalamus, bilateral occipital thalamus, bilateral caudal temporal thalamus, and bilateral lateral pre‐frontal thalamus (Figure S1), as the regions of interest (ROI). A seed reference time course was obtained within each ROI. Correlation analyses were conducted on the seed reference and the whole brain in a voxel‐wise manner for each ROI. The correlation coefficients of each voxel were normalized to *Z*‐scores with Fisher's *r*‐to‐*z* transformation creating an entire brain *Z*‐score map for each ROI of each subject.

### 2.6 Statistical analysis

We used the SPSS 24.0 (Statistical Product and Service Solutions) software for demographic statistical analysis. A chi‐square test was applied to compare the gender difference between the two groups. After the normality test, the normal measurement data were expressed as mean and standard deviation. Paired *t* test was used for intra‐group comparison, and independent sample t test was used for inter‐group comparison. The measurement data of skewed distribution were expressed as median and quartile M (P25, P75). Paired Wilcoxon signed rank test was used in the group, and Mann–Whitney *U* test was used for inter‐group comparison. The significance level *α* = 0.05.

The fMRI data statistical analysis was conducted based on the statistical module of DPABI. We used a paired *t*‐test to explore the differences between PD on state and PD off state for each ROI. A two‐sample t‐test was used to examine the differences in FC between the PD off state and healthy controls, and PD on state and healthy controls for positive results after correction. All results were corrected by Gaussian Random Field theory (GRF) [voxel‐level *p* < 0.001, cluster‐level *p* < 0.003 (0.05/16)] with gray matter volume as a covariate. Finally, signals were extracted from the significant clusters between PD off state and PD on state (∆FC). Correlations were used to explore the relationships between ∆FC and ∆UPDRSIII (UPDRSIII difference between off state and on state), ∆tremor, ∆rigidity, ∆bradykinesia and ∆axial symptoms. Pearson correlation was used for correlation analysis of normal distribution data, and spearman correlation was used for correlation analysis of skewed data.

## 3 RESULTS

### 3.1 Demographic characteristics and clinical effects of dopaminergic medication

As shown in Table 1, there was no significant difference in sex (*p* = 0.903), age (*p* = 0.144), MMSE score (*p* = 0.112), or education years (*p* = 0.739) between PD patients and controls. After the acute levodopa challenge test, the UPDRS motor score was significantly improved in all PD patients. The total UPDRS III was reduced by 51% [*z* = −5.24, *p* < 0.001]. Furthermore, symptom‐specific analyses showed that tremor reduced by 57% [*z* = −4.81, *p* < 0.001], rigidity by 54% [*t* = 10.06, *p* < 0.001], bradykinesia by 55% [*t* = 11.36, *p* < 0.001], axial symptom by 28% [*z* = −3.94, *p* < 0.001] (Table 2).

### 3.2 Functional connectivity

We mainly focused on comparing PD on state and off state to investigate the effect of dopaminergic treatment on thalamic FC in PD patients. Three subregions of the thalamus showed significant differences in the functional connectivity between PD on state and PD off state, including the right pre‐motor thalamus, the left lateral pre‐frontal thalamus, and the right lateral pre‐frontal thalamus. The other subregions showed no significant difference between PD on state and PD off state. The details are as follows:

#### 3.2.1 Right pre‐motor thalamus

**FIGURE 1:**
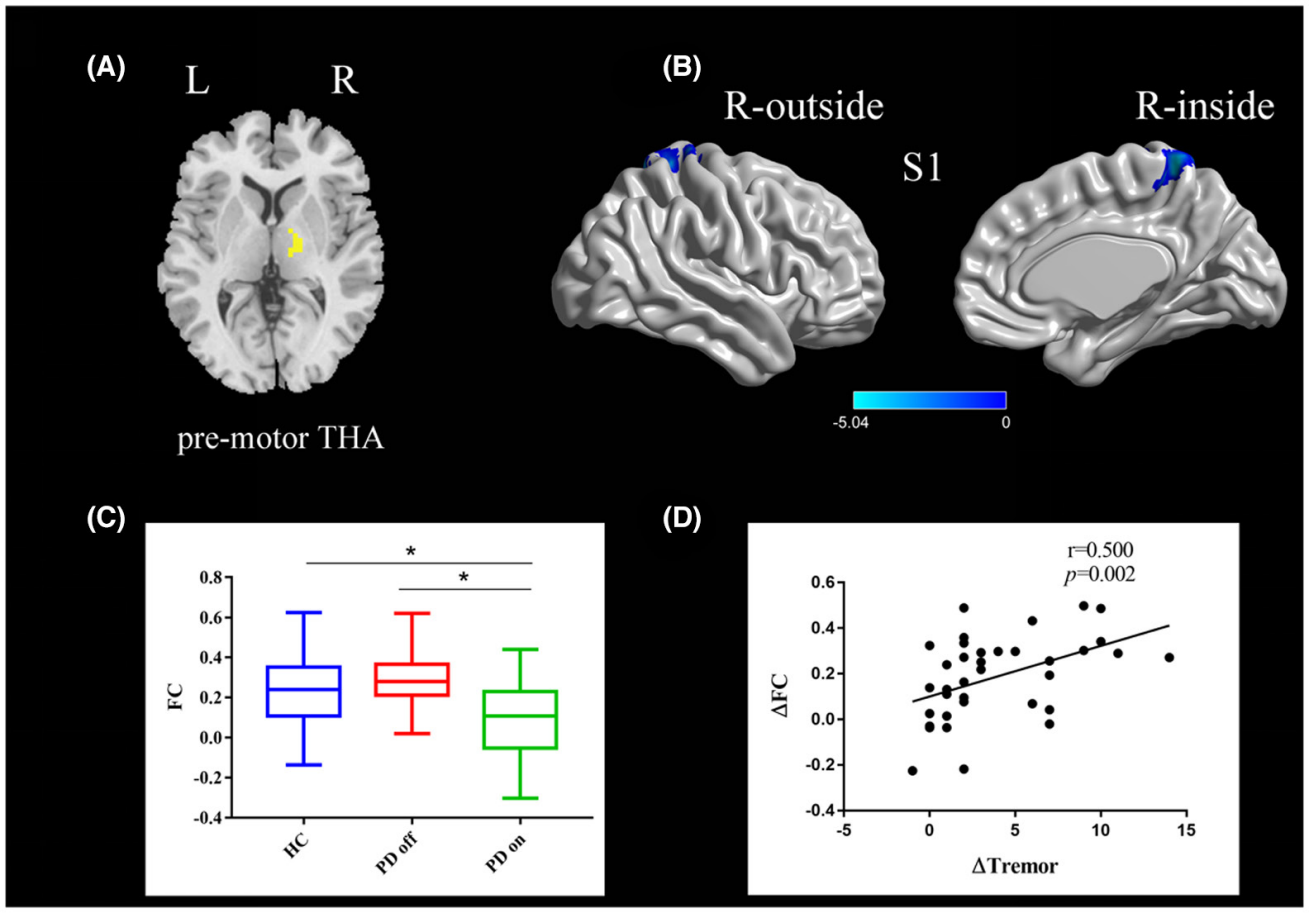
(A) The location of the right pre‐motor thalamus (yellow color) based on the Brainnetome Atlas template; (B) Brain region postcentral gyrus (S1) showing a significant difference in functional connectivity with the right pre‐motor thalamus between PD on state and off state (paired *t*‐test, voxel‐level *p <* 0.001, cluster‐level *p <* 0.003, GRF correction); the cold color indicates decreased functional connectivity in PD on state compared with PD off state (PD on < PD off); a cold color bar represents the *t* value of the paired *t* test; (C) FC value histogram for the S1 in the three groups (HC, PD off, PD on); (D) ΔFC between the S1 and right pre‐motor thalamus showing a positive correlation with Δtremor scores (spearman correlation). S1, somatosensory cortex (postcentral gyrus); L/R, left/right.* represents a statistically significant difference between the two groups.

One‐sample t tests were conducted on the *z*‐maps of each of the three groups respectively (Figure S2).The PD on state exhibited decreased functional connectivity between the right pre‐motor thalamus and the postcentral gyrus compared to the PD off state (paired *t*‐test, voxel‐level *p* < 0.001, cluster‐level *p* < 0.003, GRF‐corrected; Figure 1B and Table 3). Compared to healthy controls, PD off state exhibited decreased functional connectivity between the right pre‐motor thalamus and the left cerebellum posterior lobe (two‐sample *t*‐test, voxel‐level *p* < 0.001, cluster‐level *p* < 0.003, GRF‐corrected; Figure S1A). The PD on state exhibited decreased functional connectivity between the right pre‐motor thalamus and the left precuneus lobe (two‐sample *t*‐test, voxel‐level *p* < 0.001, cluster‐level *p* < 0.003, GRF‐corrected; Figure S5 and Table 3).

**TABLE 3:** Group difference in specific subregions of the thalamus connectivity.

ROI	Brain region	MNI coordinates			*T*‐value	Cluster size (voxels)
		*x*	*y*	*z*		
**R Pre‐motor thalamus**						
PD on state < off state	R Sensorimotor cortex	24	−33	72	−5.61	186
PD off state < HC	L Cerebellum posterior lobe	−27	−75	−48	−5.75	359
PD on state < HC	R Precuneus lobe	21	−78	51	−5.70	288
**R Lateralpre‐frontal thalamus**						
PD on state < off state	R Sensorimotor cortex	12	−48	66	−5.01	82
**L Lateralpre‐frontal thalamus**						
PD on state < off state	right paracentral lobule	−3	−24	57	−5.32	133

Abbreviations: L, left; MNI, Montreal Neurological Institute; R, right.

#### 3.2.2 Right lateral pre‐frontal thalamus

**FIGURE 2:**
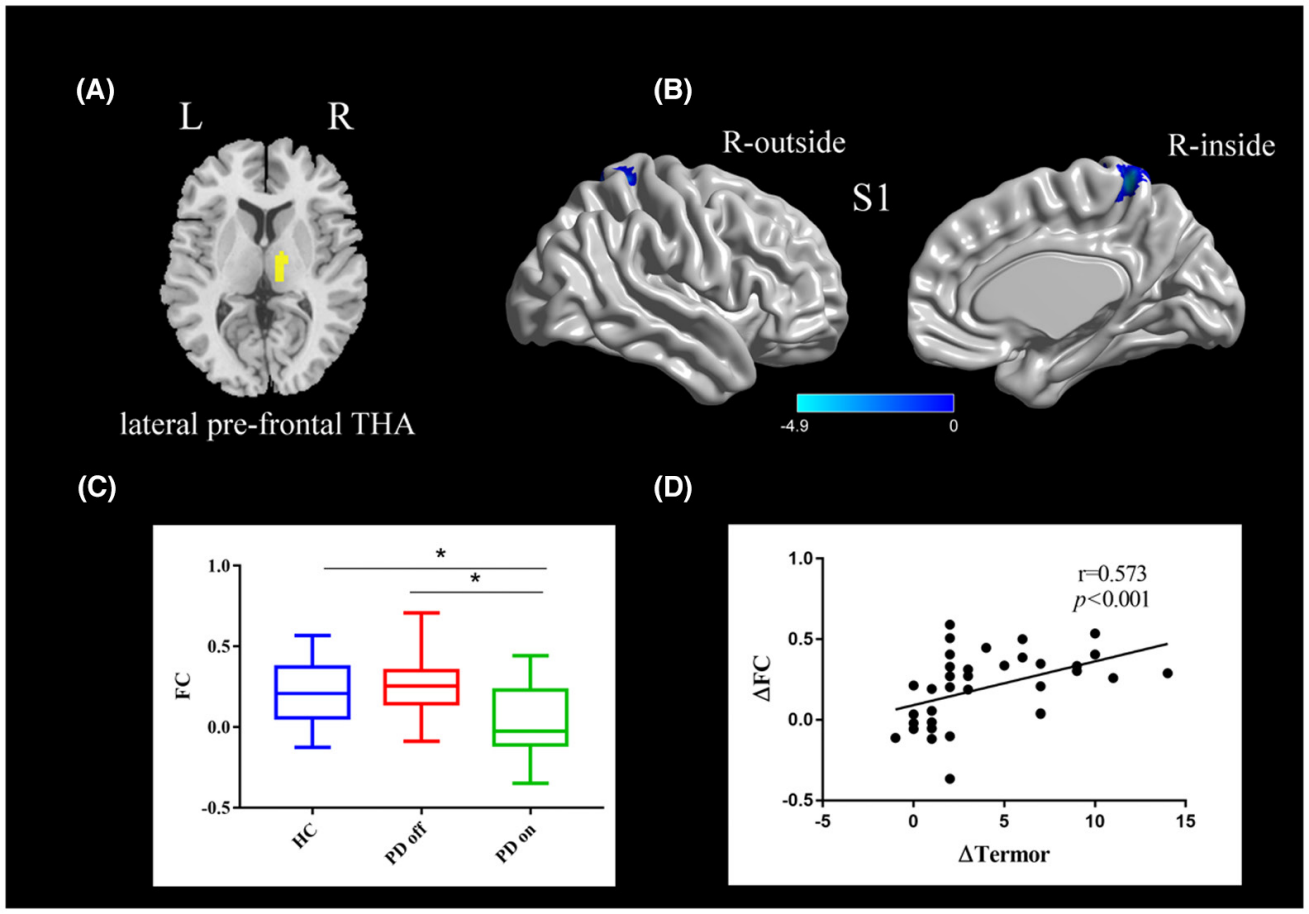
(A) The location of the right lateral pre‐frontal thalamus (yellow color) based on the Brainnetome Atlas template; (B) Brain region postcentral gyrus (S1) showing a significant difference in functional connectivity with right lateral pre‐frontal thalamus between PD on state and off state (paired *t*‐test, voxel‐level *p <* 0.001, cluster‐level *p <* 0.003, GRF correction); the cold color indicates decreased functional connectivity in PD on state compared with PD off state (PD on < PD off); a cold color bar represents the *t* value of the paired *t* test; (C) FC value histogram for the S1 in the three groups (HC, PD off, PD on); (D) ΔFC between the S1 and right lateral pre‐frontal thalamus showing a positive correlation with Δtremor scores (spearman correlation). S1, somatosensory cortex (postcentral gyrus); L/R, left/right. * represents a statistically significant difference between the two groups.

One‐sample *t* tests were conducted on the *z*‐maps of each of the three groups respectively (Figure S3).Compared to PD off state, PD on state exhibited decreased functional connectivity between the right lateral pre‐frontal thalamus and the postcentral gyrus (paired *t*‐test, voxel‐level *p* < 0.001, cluster‐level *p* < 0.003, GRF‐corrected; Figure 2B and Table 3). Compared with healthy controls, PD off state and on state exhibited no significant difference in the functional connectivity with the right lateral pre‐frontal thalamus (two‐sample *t*‐test, voxel‐level *p* < 0.001, cluster‐level *p* < 0.003, GRF‐corrected).

#### 3.2.3 Left lateral pre‐frontal thalamus

**FIGURE 3:**
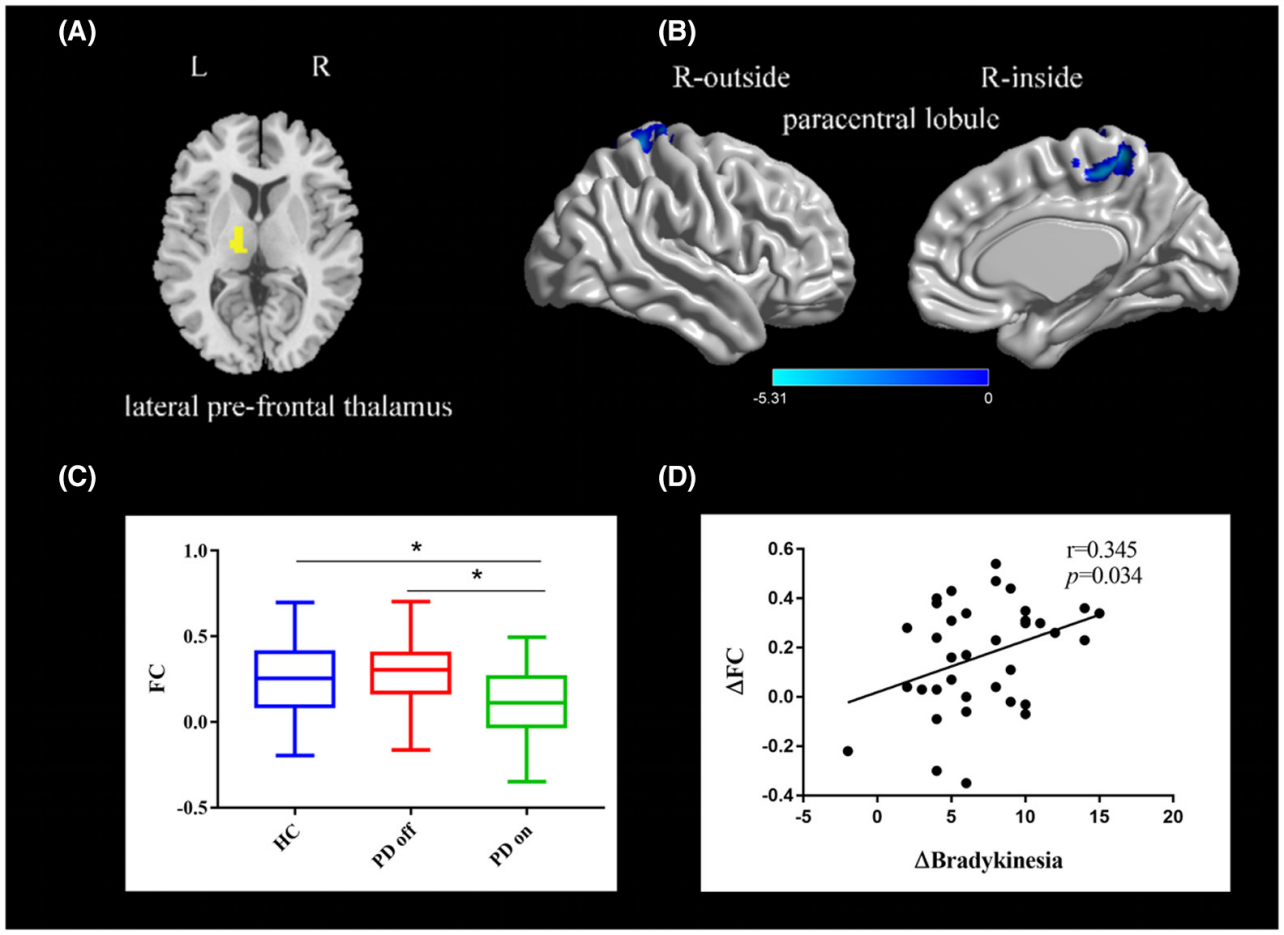
(A) The location of the left lateral pre‐frontal thalamus (yellow color) based on the Brainnetome Atlas template; (B) Brain region (right paracentral lobule) showing a significant difference in functional connectivity with left lateral pre‐frontal thalamus between PD on state and off state (paired *t*‐test, voxel‐level *p <* 0.001, cluster‐level *p <* 0.003, GRF correction); the cold color indicates decreased functional connectivity in PD on state compared with PD off state (PD on < PD off); a cold color bar represents the *t* value of the paired *t* test; (C) FC value histogram for the right paracentral lobule in the three groups (HC, PD off, PD on); (D) ΔFC between the right paracentral lobule and left lateral pre‐frontal thalamus showing a positive correlation with Δbradykinesia scores (pearson correlation). L/R, left/right. * represents a statistically significant difference between the two groups.

One‐sample t tests were conducted on the *z*‐maps of each of the three groups respectively (Figure S4).Compared to the PD off state, PD on state exhibited decreased FC between the left lateral pre‐frontal thalamus and right paracentral lobule (paired t‐test, voxel‐level *p* < 0.001, cluster‐level *p* < 0.003, GRF‐corrected; Figure 3B and Table 3). Compared to healthy controls, PD off state and on state exhibited no significant difference in the functional connectivity with the left lateral pre‐frontal thalamus (two‐sample *t*‐test, voxel‐level *p* < 0.001, cluster‐level *p* < 0.003, GRF‐corrected).

### 3.3 Correlation analysis

A correlation analysis showed that ∆tremor were positively correlated with the ∆FC between (1) the postcentral gyrus and the right pre‐motor thalamus (*r* = 0.500, *p* = 0.002; Figure 1D), (2) the postcentral gyrus and the right lateral pre‐frontal thalamus (*r* = 0.573, *p* < 0.001; Figure 2D); ∆bradykinesia were positively correlated with the ∆FC between the left lateral pre‐frontal thalamus and right paracentral lobule (*r* = 0.353, *p* = 0.034; Figure 3D).

## 4 DISCUSSION

We examined the FC changes in the 16 subregions of the thalamus under the effect of levodopa in a cohort of PD patients using a seed‐based analysis. The results showed that levodopa treatment decreased FC between (1) the right pre‐motor thalamus and the right sensorimotor cortex, (2) the right lateral pre‐frontal thalamus and the right sensorimotor cortex, and (3) the left lateral pre‐frontal thalamus and right paracentral lobule. In addition, there were significant correlations between the observed decreased FCs and the improvement of clinical symptoms.

Our results are consistent with a previous study that reported a decreased FC between the thalamus and the sensorimotor cortex in advanced PD patients after the levodopa challenge test.^26^ Moreover, the decreased FC was associated with greater improvement in UPDRS‐III scores. Another fMRI study found an increased FC between the thalamus and the sensorimotor cortex in PD off state.^27^ Besides, a magnetoencephalogram (MEG) study also found a significant decrease in cerebro‐cerebral coupling between thalamus and cortical motor areas after levodopa intake in tremor‐dominant PD patients.^28^ However, the above studies did not explore the function of thalamic subregions. Therefore, we made a more detailed division of the thalamus and found that the right pre‐motor thalamus and the right lateral pre‐frontal thalamus were associated with the clinical tremor symptom.

Studies have shown that the underlying pathological mechanisms of tremor may differ from those of bradykinesia and rigidity in the classic triad of PD symptoms.^29^ The classic BGMC circuit is a good explanation for bradykinesia and rigidity but not for tremor. In addition, Helmich et al. (2011) proposed that resting tremor is associated with a distinct cerebellothalamic circuit involving the VIM, motor cortex (MC), and cerebellum (CBLM).^8^ The VIM, as one of the crucial nodes in the tremor circuit, is involved in modulating resting tremor amplitude and in processing tremor‐related afferents.^7^, ^30^ However, traditional MR and CT images cannot identify VIM, and its precise location is determined by individual stereotactic coordinates or population‐based stereotactic coordinates.^31^ This study found that the right pre‐motor thalamus subregion contains VIM in anatomical location. The right pre‐motor thalamus could also improve tremor symptoms in the tremor circuit by reducing the FC with the sensorimotor cortex under levodopa which is similar in function to VIM. Therefore, we speculate that the right pre‐motor thalamus is highly coincident with VIM, providing us a new understanding of the thalamic subregions. Meanwhile, VOP is an another key node which participated in the tremor circuit. It receives GPi projection and connects the basal ganglia to the VIM–MC–CBLM circuit via MC.^8^ Calculations showed that the right lateral pre‐frontal thalamus partially overlapped with VOP. These findings provide new evidence for the importance of thalamus subregions in the tremor circuit of PD.

In addition to the primary motor cortex, the primary somatosensory cortex is also considered part of the MC. Motor behavior, including planning, execution, and controlling, is a complex neural process dependent on the correct sampling of multiple sensory modalities from the body's periphery and external environment. Motor outputs are abnormal and inaccurate without correct processing and translation of sensory input before or during movement. There is a close link between sensory processing and movement production.^32^ Meanwhile, a previous study proposed that motor disturbances are superimposed on an abnormal ‘background noise’ in sensory processing, hindering sensorimotor integration and ultimately resulting in the appearance of clinical motor symptoms in PD.^33^ According to previous studies, the somatosensory cortex is closely associated with parkinsonian tremor.^27^, ^34^ Another study reported that VIM participates in voluntary action and relays afferent input to the somatosensory area.^35^, ^36^ Increased imaging‐related activity in the somatosensory cortex of patients was detected using a controlled motor imagery task during fMRI scanning after comparing tremor‐dominated PD patients with healthy controls and patients with non‐tremor PD.^37^ The study also found that tremor‐related activity was localized to the motor cortex, cerebellum, and (VIM). Therefore, this study suggests that Parkinson's tremor affects motor representation through VIM to modulate central somatosensory processing, which may explain the clinical difference between tremor and non‐tremor PD patients. These results are consistent with our present study, which emphasizes the critical role of the somatosensory (S1) in the pathological mechanism of PD. Our findings suggest that the FC between the thalamus and the sensorimotor area in PD patients increased aberrantly in the off state compared with the HC controls. Levodopa administration can significantly reverse the abnormal changes and improve tremor symptom.

This study also found that the improved bradykinesia in PD was positively correlated with the ∆FC between the left lateral pre‐frontal thalamus and right paracentral lobule in the on state versus off state. This further confirms that the thalamus participates in the tremor circuit and plays an important role in the classic BGMC circuit. A previous study reported a significantly lower FC between the paracentral lobule and subthalamic nucleus after a moderate dose of levodopa in PD patients, which is associated with better symptom improvement in PD.^26^ These findings were partly consistent with our results. Therefore, we speculate that the paracentral lobule may be an important potential target of levodopa in treating PD, especially with the subtype of bradykinesia.

The PD off state exhibited decreased functional connectivity between the right pre‐motor thalamus and the left cerebellum posterior lobe compared to healthy controls. The mechanisms underlying the pathophysiology and treatments have traditionally focused on the basal ganglia‐thalamo‐cortical pathways due to striatal dopamine loss, but recent evidence has highlighted the role of the cerebellum.^38^, ^39^, ^40^ Disparate cerebellar lobules are associated with dissociable “cognitive” and “motor” networks.^41^ The motor cerebellum includes the lobules V, VI, VIIb, and VIII projecting to motor regions (pre‐ and post‐central gyrus), and the cognitive cerebellum includes Crus I and II projecting to pre‐frontal and parietal cortices.^42^, ^43^, ^44^ A previous animal study using macaque Monkeys showed that the cerebellum has a strong disynaptic projection to the striatum by the thalamus and may influence the pathways involved in basal ganglia processing.^45^, ^46^ Subsequently, Wu et al.^47^ investigated the effective connectivity of the brain networks while performing self‐initiated movements in patients with bradykinesia /rigidity PD. The study found that the connectivities between cortico‐cerebellar motor regions were strengthened, whereas the striatum–cerebellar connectivities were weakened. Similarly, tremor patients displayed decreased FC between the VIM and the cerebellum and increased FC between VIM and the motor cortex. Furthermore, tremor severity correlated with these FC changes.^48^ These findings are partly consistent with this study, suggesting that the FC between the thalamus and cerebellum may be reduced in PD patients. In the hypoactive cortico‐striatal motor circuitry context, typically seen in PD, we speculate that the changes in cortico‐cerebellar motor circuitry may exert pathological manifestations of PD or a compensatory effect.

There are some limitations in the present study. First, the study was based on a standardized acute levodopa challenge test, but the levodopa dose was excessive compared to the daily dose of the drug in PD patients. Therefore, the effect of a normal dose levodopa intervention on FC in the subregions of the thalamus in PD patients is still worth further investigation. Second, only the short‐term changes in the thalamus subregions under levodopa administration were observed, neglecting the effects of long‐term levodopa interventions. The responsiveness of PD patients to levodopa could gradually decrease as PD progresses. Changes in the FC of the subthalamic regions may help us evaluate the responsiveness of patients to levodopa treatment. Finally, different brain templates may lead to different results. The Brainnetome atlas template used in this study is based on differences in the structural connection patterns of each voxel [diffusion tensor imaging (DTI) fiber tracking] and the aggregation voxel with the same connection patterns to complete the definition of brain region boundaries by a clustering algorithm (Fan et al.; Paxinos et al.). It makes our results more reliable and has been cited and verified in many previous studies.^49^, ^50^

## 5 CONCLUSION

This study demonstrated that levodopa treatment in PD decreases the FC between the right pre‐motor thalamus and the right somatosensory cortex, the right lateral pre‐frontal thalamus and the right somatosensory cortex, and the left lateral pre‐frontal thalamus and right paracentral lobule. Additionally, the beneficial effect of levodopa on tremor and bradykinesia is related to the decreased FC of the tremor circuit and BGMC circuit, respectively. Our results provide a basis for further exploration of the functional activity of thalamic subregions and new insights into the precision treatment in PD patients.^51^

## AUTHOR CONTRIBUTIONS

Liu Weiguo and Yu Miao: designed the study. Zhong Yuan, Sun Yu, Yang Jiaying, Zhang Wenbin and Yan Lei: collected the data. Liu Wan and Shen Yang: analyzed the data and prepared the manuscript.

## FUNDING INFORMATION

This work was supported by the National Natural Science Foundation of China (NSFC) (Nos. 81571348, 81701675, 81903589, 81701671), the National Key Research and Development Program of China (2017YFC1310300, 2017YFC1310302, and 2016YFC1306600), the Key Project supported by Medical Science and technology development Foundation, Nanjing Department of Health (No. JQX21006), the Science and Technology Program of Jiangsu Province (Nos. BE2019611, BE2018608), the Jiangsu Provincial Natural Science Foundation of China (BK20151077).

## CONFLICT OF INTEREST STATEMENT

The authors declare that they have no competing financial interests.

## Supporting information


Figure S1‐S5

## Data Availability

The data that support the findings of this study are available on request from the corresponding author. The data are not publicly available due to privacy or ethical restrictions.
