# Supplementary material for: Levodopa improved different motor symptoms in patients with Parkinson's disease by reducing the functional connectivity of specific thalamic subregions
Source: CNS Neurosci Ther. 2023 Jul 14;30(2):e14354. doi: 10.1111/cns.14354 (PMC10848087; doi:10.1111/cns.14354)
Supplement: Supplementary file 1 — Figure S1‐S5 [file CNS-30-e14354-s001.docx]

# Supplementary Material


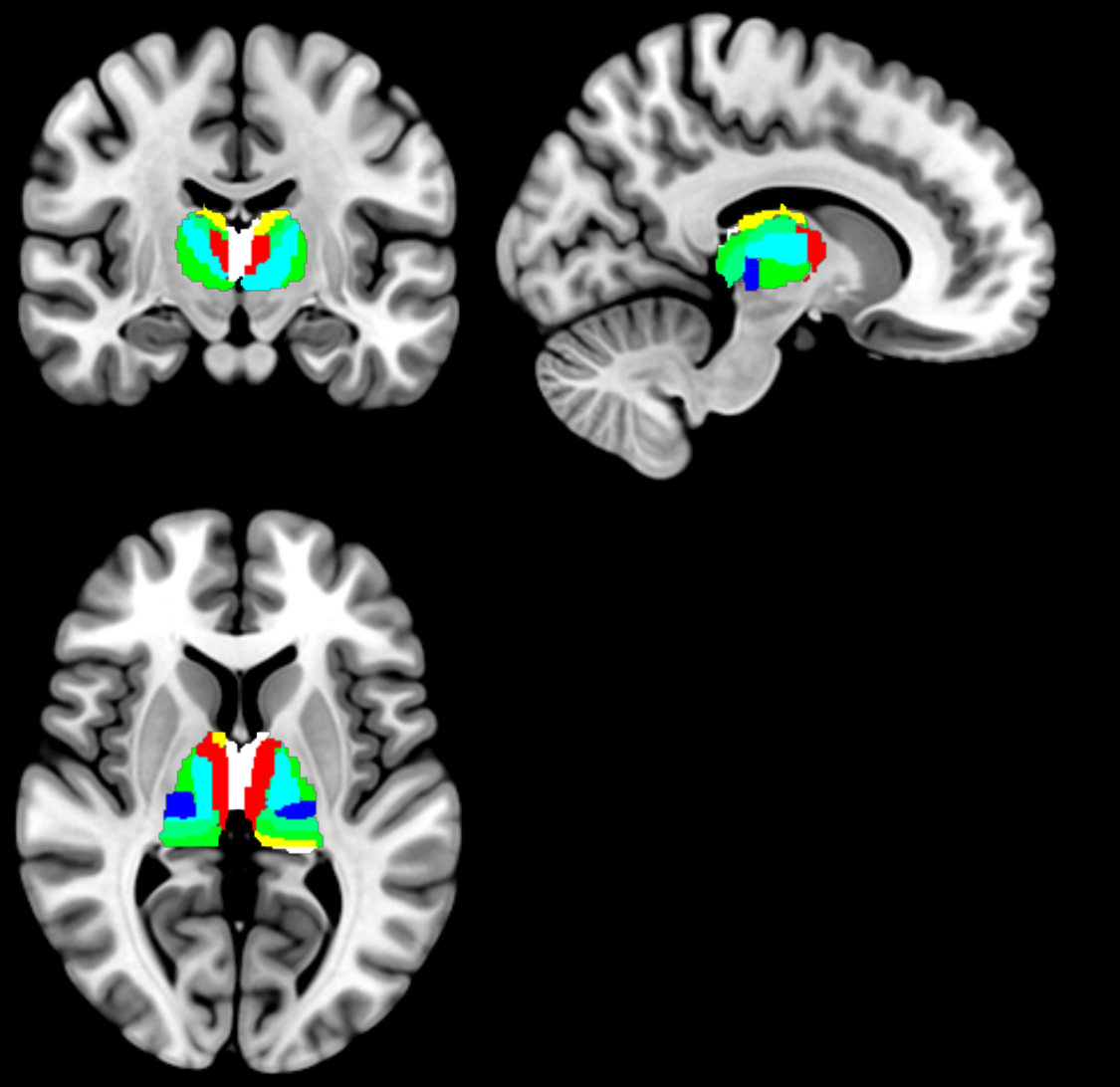


**Figure S1** 16 subregions of the thalamus from the Human Brainnetome Atlas on the coronal, sagittal and cross sections.


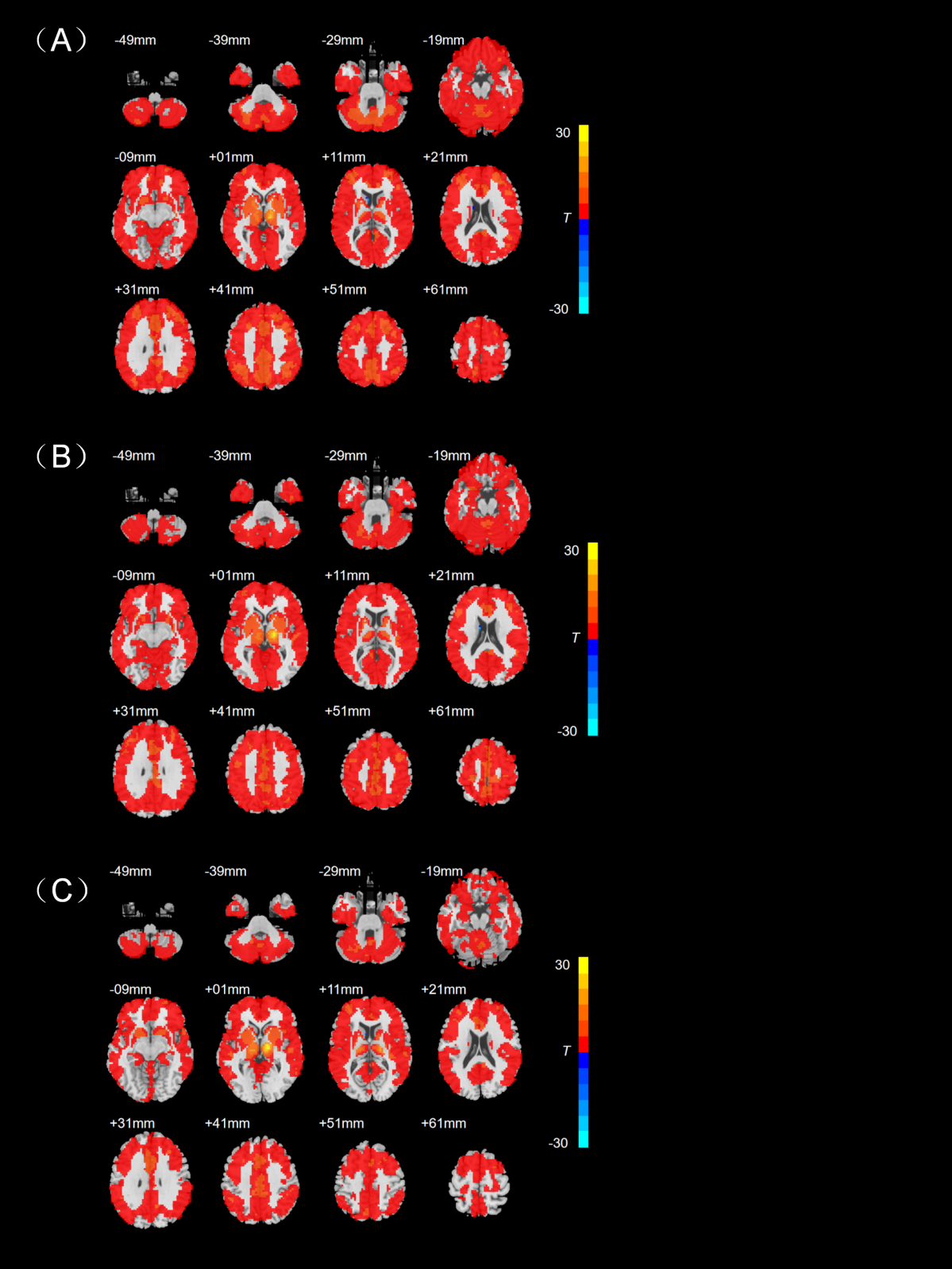


**Figure S2** (A) Functional connectivity pattern of right pre-motor thalamus in healthy controls (one-sample t-test, voxel-level P < 0.001, cluster-level P < 0.003, GRF-corrected); (B) Functional connectivity pattern of right pre-motor thalamus in PD off state (one-sample t-test, voxel-level P < 0.001, cluster-level P < 0.003, GRF-corrected); (C) Functional connectivity pattern of right pre-motor thalamus in PD on state (one-sample t-test, voxel-level P < 0.001, cluster-level P < 0.003, GRF-corrected).


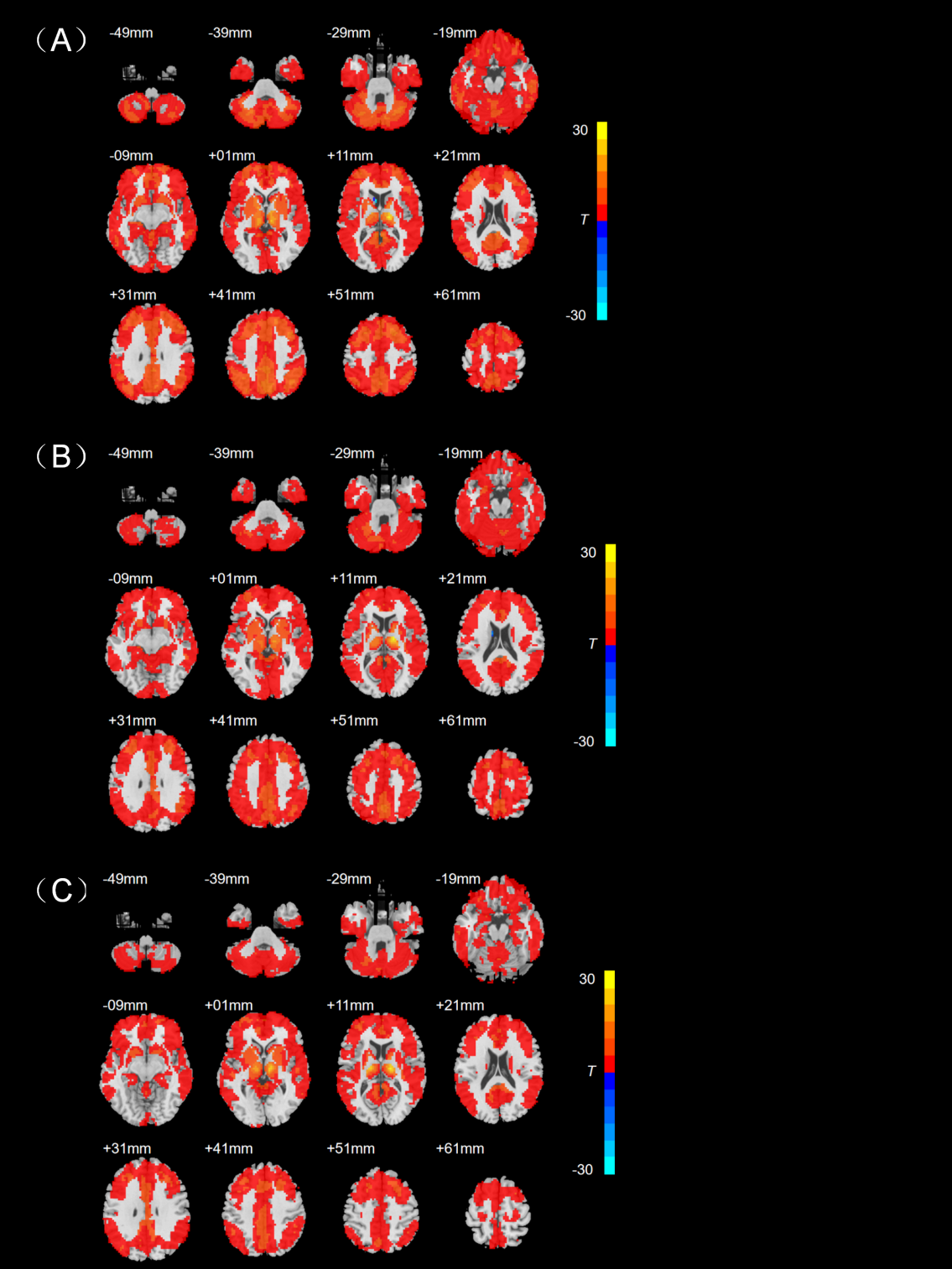


**Figure S3** (A) Functional connectivity pattern of right lateral pre-frontal thalamus in healthy controls (one-sample t-test, voxel-level P < 0.001, cluster-level P < 0.003, GRF-corrected); (B) Functional connectivity pattern of right lateral pre-frontal thalamus in PD off state (one-sample t-test, voxel-level P < 0.001, cluster-level P < 0.003, GRF-corrected); (C) Functional connectivity pattern of right right lateral pre-frontal thalamus in PD on state (one-sample t-test, voxel-level P < 0.001, cluster-level P < 0.003, GRF-corrected).


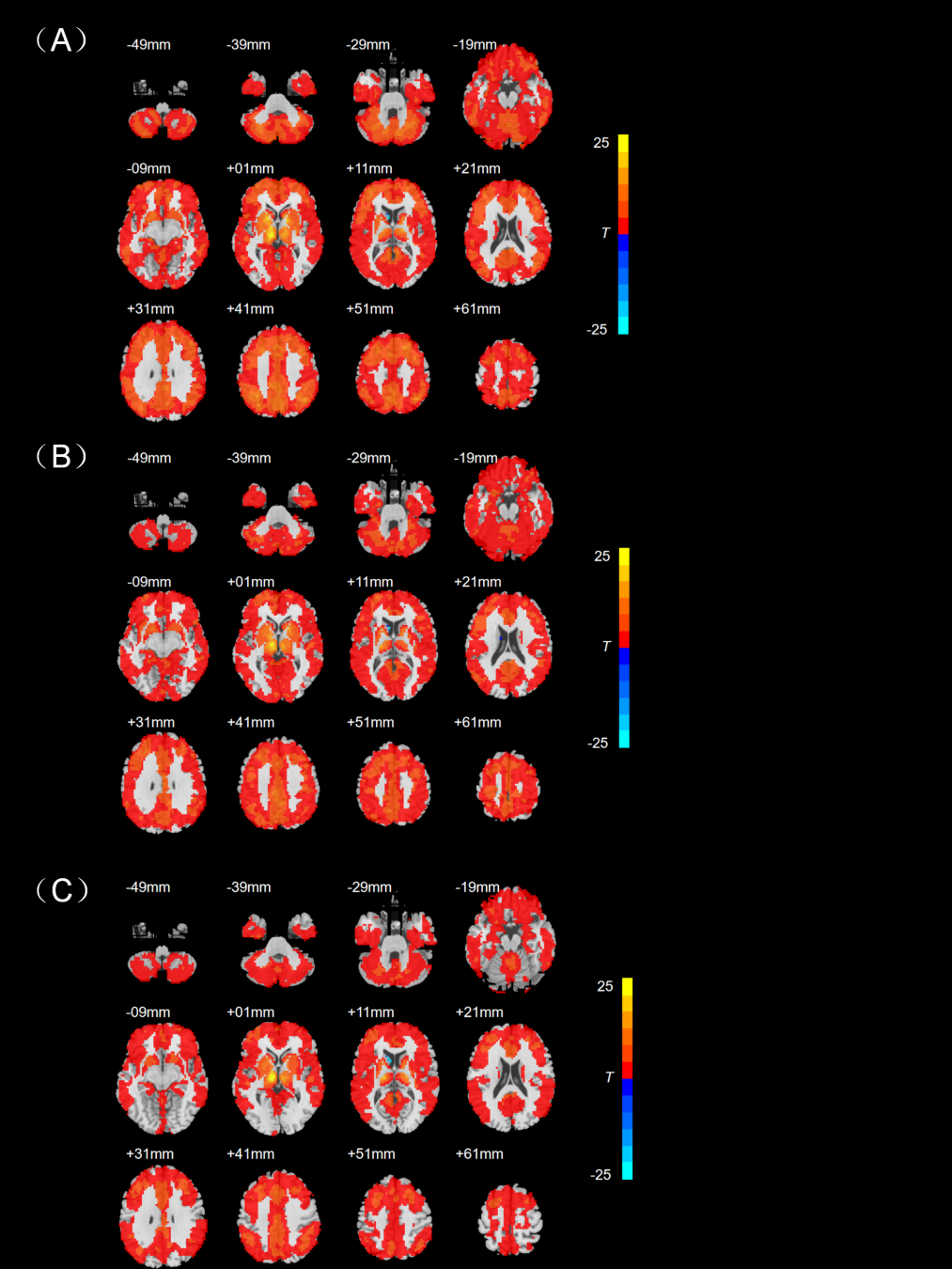


**Figure S4** (A) Functional connectivity pattern of left lateral pre-frontal thalamus in healthy controls (one-sample t-test, voxel-level P < 0.001, cluster-level P < 0.003, GRF-corrected); (B) Functional connectivity pattern of left lateral pre-frontal thalamus in PD off state (one-sample t-test, voxel-level P < 0.001, cluster-level P < 0.003, GRF-corrected); (C) Functional connectivity pattern of left lateral pre-frontal thalamus in PD on state (one-sample t-test, voxel-level P < 0.001, cluster-level P < 0.003, GRF-corrected).

**
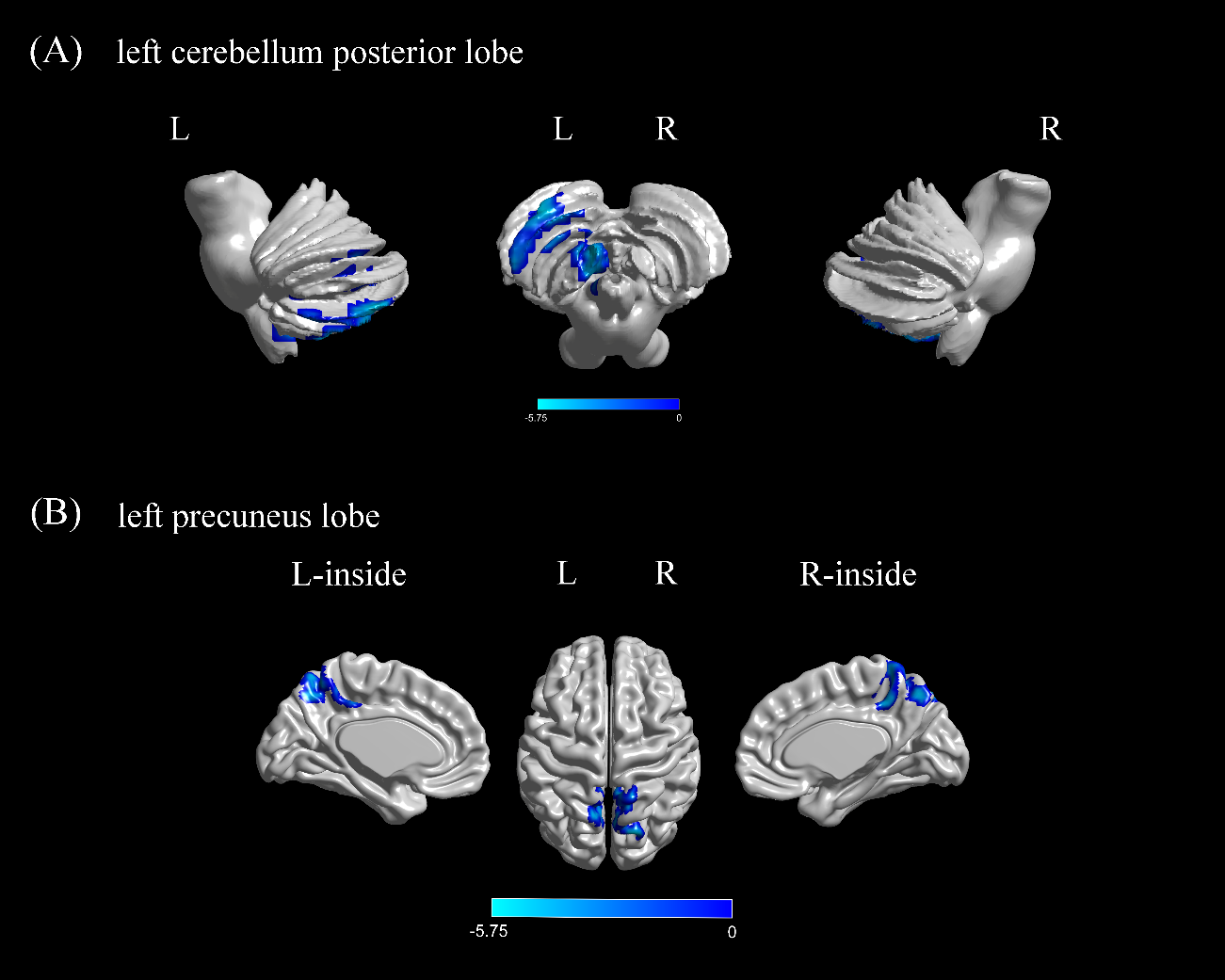
**

**Figure S5** (A) Left cerebellum posterior lobe; the cold color indicates decreased functional connectivity in PD off state compared with HC (PD off < HC); (B) Left precuneus lobe; the cold color indicates decreased functional connectivity in PD on state compared with HC (PD on < HC).
